# A Systematic Review on Tibial Cortex Transverse Transport in the Treatment of Ischemic Ulcers of the Lower Limb

**DOI:** 10.1177/10711007251341312

**Published:** 2025-06-26

**Authors:** Abby Luxon, Anxhela Syziu, William D Harrison, Amirul Islam, Lyndon Mason

**Affiliations:** 1 University of Liverpool, Liverpool, United Kingdom; 2 Liverpool University Hospitals NHS Foundation Trust, Liverpool, United Kingdom

**Keywords:** tibial cortex transverse transport, TTT, diabetes, ischemic ulcers, treatment of ischemic ulcers, ischemic ulcers of the lower limb, lower limb

## Abstract

**Background:**

Ischemic foot ulcers are a debilitating but common finding from many different conditions and can result in severe and life-threatening complications such as amputation and death. In recent years, tibial cortex transverse transport (TTT) has been used to treat ischemic ulcers with the aim to improve revascularization and wound healing. Our aim with this systematic review is to analyze the current literature to study the effects this surgery has on the treatment of ischemic ulcers of the lower limb.

**Methods:**

A search was conducted using the 3 databases Web of Science, PubMed, and Scopus to collate all articles associated with the use of TTT in the treatment of lower limb ischemic ulcers. Duplicate articles were removed, and the remaining articles were filtered and screened using set inclusion criteria such as patients with a diagnosed foot ulcer of ischemic origin, patients treated with TTT alone or combined with other techniques on the affected leg, unilateral or bilateral ischemic ulcers, studies that had evidence of treatment outcomes related to ulcer such as visual analog scale (VAS), ankle-brachial index (ABI) measurements, ulcer healing rate and time, recurrence rate, and complications. The exclusion criteria consisted of duplicated studies, overlapping data in studies, TTT used to treat nonulcerous conditions, other systematic reviews, articles with publication language other than English, no access to full text of article, and case reports.

**Results:**

A total of 13 articles were included in the final selection, involving 924 patients, with 724 treated with TTT, in which 701 were diabetic patients. The results extracted demonstrated improvements in healing rates and times, vascular endothelial growth factor, ABI scores, VAS scores, and limb salvage. With regard to the complications and risks, pin-site infections and tibial fractures were infrequent and treated quickly.

**Conclusion:**

Overall, the use of TTT has been associated with unusual success in improving revascularization and healing times in treating ischemic ulcers of the lower limb resulting in better outcomes for the patient and may provide a potential alternative treatment to the more conventional, widespread treatments currently used in clinical practice. Tibial fractures and pin-site infections are relatively rare complications that have been reported with use of this treatment.

## Graphical abstract

**Graphical Abstract:**
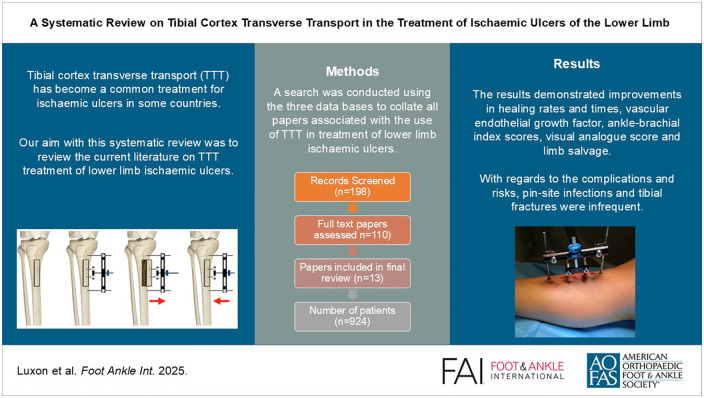
This is a visual representation of the abstract.

## Introduction

Ischemic ulcers of the lower limb can be associated with a range of conditions, such as diabetes, thromboangiitis obliterans, vasculitis, and peripheral arterial disease.^23^ Ulceration can occur alone or in combination with other clinical manifestations.^28^ The current accepted treatment for ischemic ulcers is dependent on severity and type of ulcer, ranging from lifestyle changes and nonsurgical treatments to surgical interventions for the most severe presentation of ischemic ulcers.^4^ These conventional treatments, such as bypass grafting surgery, often consist of focusing on the macrovascular areas of ischemic ulcers. Recent literature demonstrates that although correction of large vascular blood flow can benefit ischemic ulcers, without a way to address microvascular pathology, ulcers may recur or fail to heal, resulting in complications.^3^

With an increasing incidence of ischemic ulceration, the search for better outcomes for ischemic ulcers is paramount, with the overall aim being to improve healing times, prevent recurrence, and prevent complications such as amputations.^30^ The first published use of tibial cortex transverse transport (TTT) for the treatment of ischemic ulceration was in 2001 to treat thromboangiitis obliterans.^25^ This first application of TTT, triggered a cascade of studies and investigations into this surgery and was applied to many major lower limb ischemic diseases. The most noteworthy being the research and studies focusing into ischemic limb ulcers as a complication of diabetes. The surgery itself, a modified version of the well-established Ilizarov technique, is used to increase angiogenesis and subsequently the regeneration of skin.^16^

TTT can be achieved using a specially designed unilateral external fixator or standard Ilizarov apparatus. For simplicity, commercially available tailor-made external fixator technique is summarized here. This fixator has a linking device permitting the corticotomy fragment to gradually distract and compress by turning a knob. Two half pins with linking device are screwed into the corticotomy fragment for distraction while 2 other half pins are screwed into the tibial shaft proximal and distal to the fragment for constructing the external fixator.^14^

**Figure 1.**
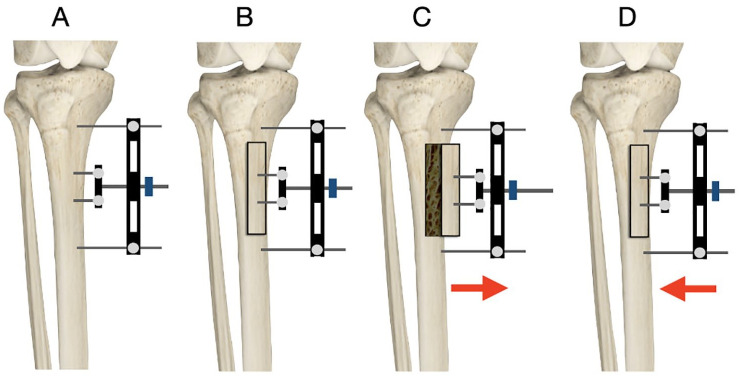
Illustration of technique. (A) The fixator device is applied to the medial side of the tibia with 2 half pins screwed into the planned corticotomy area (over the anteromedial part of tibial metaphysis usually 3-5 cm below the tibial tuberosity), and another 2 half pins placed above and below the planned fragment. (B) A corticotomy is performed as planned, to create a rectangular cortical bone window measuring about 5 × 1.5 cm. (C) After a period of 5 days, the corticotomy fragment is gradually moved transversely outward, with a speed of 1 mm/d for 10 days using the external fixator. (D) After waiting 3 days after the 10 mm of distraction has been achieved, compression is started at the same speed for the next 10 days to move the fragment back to the initial position. Two weeks later, the fixator is removed.

**Figure 2.**
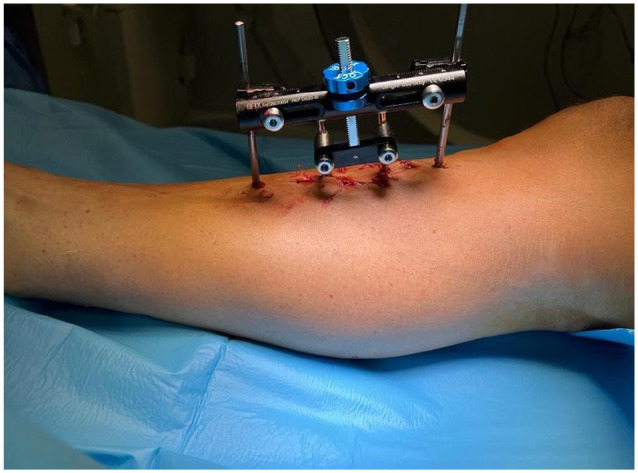
Clinical photograph after application of a specially designed TTT system. Courtesy of Cybion (www.cybion.de).

Under anesthesia, skin incision is made for corticotomy site over the anteromedial part of tibial metaphysis usually 3-5 cm below the tibial tuberosity. Corticotomy is performed to create a rectangular cortical bone window measuring about 5 × 1.5 cm by multiple drill holes taking precaution to avoid thermal necrosis. A thin and sharp osteotome is used to perform subperiosteal osteotomy by breaking the bone bridges between the drill holes. The bone flap is then gradually moved transversely outward after 5 days of corticotomy with a speed of 1 mm/d for 10 days. After waiting 3 days, compression is started at the same speed for the next 10 days to move the fragment back to the initial position. Two weeks later, the fixator is removed. An illustration of the TTT technique is shown in Figure 1 and an image example of the specialized TTT system is shown in Figure 2.^14^

The history behind the development of this surgery came from Ilizarov’s study in 1989, where Ilizarov was investigating osteogenesis and changes in soft tissue during limb lengthening on canine tibias.^12^ This article investigated not only longitudinal lengthening but also the transverse distraction model, which is the technique now used in TTT. Ilizarov’s initial focus during this study was on osteogenesis itself; however, he found the capillary network regenerated and blood flow increased.^12,16^ A study undertaken a few years later, focusing on revascularization in distraction osteogenesis, demonstrated that blood flow increased up to nearly 10 times the control canine limb peaking at 2 weeks then lowering to around 4 or 5 times the control during the rest of the distraction. During the consolidation period, 2 to 3 times more blood flow was found in comparison to the control limb, demonstrating the benefits of corticotomy in increasing blood flow.^2^

From here the technique of TTT has developed and clinical applications have moved to treatments of ischemic diseases of the lower limb, and findings are indicative of blood vessel and tissue regeneration. In one study, improvements in wound healing, visual analog scale (VAS), ankle-brachial index (ABI), expression of angiogenesis factors, and a lack of ulcer recurrence were all found, indicating a positive impact of TTT on ischemic ulcers.^21^ TTT is a new and evolving research landscape. Clinical benefit and good outcomes from this surgery are reported, but there have been very little summative data.

## Aims

The aims of this systematic review are to identify the main treatment outcomes, while also analyzing the benefits and limitations of TTT. The primary outcome is focused on healing rates, with other outcomes focusing on ABI, VAS score, analysis of complications such as amputations and vascular endothelial growth factor (VEGF) outcomes.

## Methodology

A systematic search was conducted using 3 databases, Scopus, PubMed and Web of Science, between January 2024 and January 2025. There were no limitations in the search field in any of these databases, and the search terms were created by identifying key information within the decided research field, and extending to synonyms and similar terminology. It was important to focus on the specific surgery of TTT, as articles based on Ilizarov’s technique alone did not meet the criteria set out for the research question. Once all articles and records were retrieved from each database, they were extracted and transferred into Endnote 20 for further study selection.

After the articles were extracted from the aforementioned databases, each article was screened in accordance with the inclusion criteria. The inclusion criteria consisted of patients with a diagnosed foot ulcer of ischemic origin, patients treated with TTT alone or combined with other techniques on the affected leg, unilateral or bilateral ischemic ulcers, studies that had evidence of treatment outcomes related to ulcer such as VAS score, ABI measurements, ulcer healing rate and time, recurrence rate, and complications. The exclusion criteria consisted of duplicated studies, overlapping data in studies, TTT used to treat nonulcerous conditions, other systematic reviews, articles with publication language other than English, no access to full text of article, and case reports.

A title and abstract screening process then took place, and any titles or abstracts that were irrelevant to the title were then excluded and deleted. From this point, this left 57 articles to which full text was accessed and screened for eligibility for inclusion in the final article analysis. The database searches, article screenings, and selection were independently conducted by 2 reviewers, A.L. and A.S., to increase reliability and identify all the relevant articles as per Preferred Reporting Items for Systematic reviews and Meta-Analyses guidelines (PRISMA).^22^

## Results

**Figure 3.**
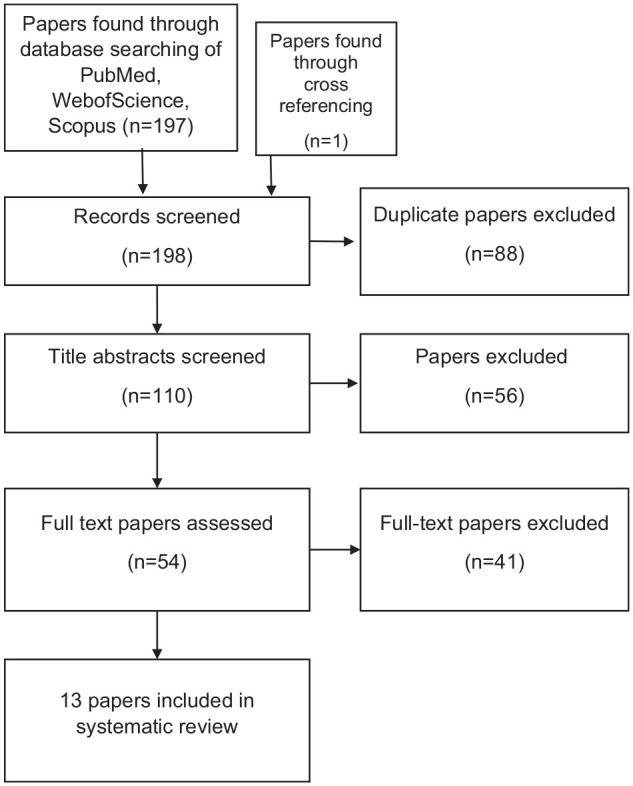
Flow chart based on the PRISMA flow diagram.^22^

The flow chart in Figure 3 represents the search and selection process of the articles. In total, 198 articles were retrieved from the 3 databases and from cross-referencing of articles. From this, 88 articles were found to be duplicates and 110 articles were then reviewed using title and abstract to determine if relevant to the decided title. A total of 56 articles were deemed irrelevant or not related to the original question and were removed from the final selection. Fifty-four articles remained and were screened for full-text eligibility, where 13 were included for analysis. From the 54 articles, reasons for exclusion included irrelevance to the study criteria and incomplete or irrelevant data to the main outcomes focused on during this systematic review.

The 13 articles were then analyzed, and key characteristics were picked out of these studies shown in Table 1. The study types consisted of case-control (n = 3), case series (n = 8), and cross-sectional (n = 2). Articles ranged from 2010 to 2024, and country of origin consisted of China (n = 12) and India (n = 1). The articles ranged in type of condition being treated with TTT including diabetic foot ulcers and thromboangiitis obliterans.

**Table 1.** Key characteristics from selected articles.

Articles	Country	Type of Study	Date of Study	Groups	Age	Total Patients	% Female	Follow-up (mo)	BMI	Ulcer Size/Classification (Wagner/Texas)	Ulcer Location	Excluded/Lost to Follow-up
Yu et al, 2024^31^	China	Cross-sectional	2024	TTT	Male 63 ± 12 Female 68 ± 14	68	28	/	N/A	Wagner Grade 3: 40 Grade 4: 28	Bilateral: 5 Unilateral: 63 (Left: 27 Right: 36)	N/A
Wen et al, 2024^29^	China	Cross-sectional	2024	TTT	48-82	52	38	12	N/A	Texas 2B: 9 2C: 5 2D: 11 3B: 10 3C: 2 3D: 14	Left foot: 27 Right foot: 25	2
Rohit et al, 2023^26^	India	Case series	2019-2021	TTT	34-70	10	10	12	N/A	N/A	Right side: 7 Left side: 3	0
Qin et al, 2023^24^	China	Case series	2017-2019	TTT	46-82	68	18	12	N/A	Texas UDFU, BDFU 2B: 6, 10 2C: 2, 1 2D: 7, 2 3B: 6, 4 3C: 1, 3 3D: 12, 14	UDFU: 34 BDFU: 34	0
Liu et al, 2023^17^	China	Case series	2020-2021	TTT	63.43±12.52	63	35	3	N/A	All Wagner ⩾grade 2 LP 15.64 ± 12.99 cm² MP 13.93 ± 14.90 cm²	N/A	3
Jianda et al, 2023^13^	China	Case series	2017-2021	TTT	63.57 ± 4.89	21	57	6	23.93 ± 2.85	67.43 ± 13.31 cm² (46-97 cm² )	Unilateral heel ulcers: 21	N/A
Ou et al, 2022^21^	China	Case series	2017-2019	TTT	67.0 ±11.9	19	53	12	21.69 (2.49; 16.89– 25.39)	N/A	Left foot: 10 Right foot: 9	1
Fan et al, 2022^8^	China	Case-control	2017-2019	TTT	42-65	21	29	14.5	23.5 ± 4.5	Wagner Grade 2: 2 Grade 3: 14 Grade 4: 4	Left foot: 12 Right foot: 9	0
				Healthy	40-64	20	25	14.5	23.7 ± 6.5	N/A	N/A	0
Chang et al, 2023^5^	China	Case series	2021	TTT	45-66	13	31	13	n/a	Wagner Grade 3: 8 Grade 5: 5	Left foot: 5 Right foot: 8	0
Yuan et al, 2021^32^	China	Case series	2016-2019	TTT	68.3 ± 7.1	201	47	12	23.53, 95%CI[23.07, 23.89]	Texas 2C: 139 2D: 36 3C: 0 3D: 26	N/A	0
Nie et al, 2021^20^	China	Case-control	2018-2019	TTT	63.5	42	21	12	N/A	16.2 cm² Texas 2B: 6 2C: 6 2D: 12 3B: 1 3C: 1 3D: 16	Foot ulcers: 35 Leg ulcers: 7	2
				Control	63.1	43	23	12	N/A	11 cm² Texas 2B: 9 2C: 7 2D: 11 3B: 2 3C: 1 3D: 13	Foot ulcers: 30 Leg ulcers: 13	1
Li et al, 2021^15^	China	Case series	2010-2019	TTT	33.8 ± 14.6	13	62	28	N/A	11.2 cm² Texas 2C: 1 2D: 4 3B: 3 3D: 5	Right-sided lesions: 10 Left-sided lesions: 3	0
Chen et al, 2020^6^	China	Case-control	2014-2017	TTT	61.0 ± 10.0	136	30	24	23 ± 3.2	44 ± 10cm²	Forefoot: 62 Midfoot: 40 Hindfoot: 20 Above ankle: 14	1
				Control	60.0 ± 11.0	137	36	24	23 ± 3.4	41 ± 9 cm²	Forefoot: 71 Midfoot: 41 Hindfoot: 14 Above ankle: 11	2

Abbreviations: BDFU, bilateral diabetic foot ulcer; BMI, body mass index; LP, learning phase; MP, mastery phase; N/A, not applicable; TTT, tibial cortex transverse transport; UDFU, unilateral diabetic foot ulcer.

In total, 924 patients were included in the 13 studies with 724 patients treated with TTT, and 701 (96.8%) of those patients were diabetic. The process of TTT varied in each article, with some using TTT alone and others combining more traditional treatments such as vacuum sealing drainage (VSD), debridement, antibiotic bone cement, and other treatments. The results and range of outcomes and findings have been represented in Table 2 and focus on different quantitative and qualitive outcomes such as ABI, VAS, healing times, healing rates, recurrence rates, measurement of VEGF and SDF-1, claudication distances, and death reduction. A list of complications was also collated, and are summarized in Table 3.

**Table 2.** Cross-Tabulation of Articles and Outcomes.

Outcomes	Ulcer Healed	Healing Time, mo	ABI		VAS		Amputation Rates	VEGF (pg/mL)	
			Preop	Postop	Preop	Postop		Preop	Postop
Yu et al, 2024^31^	63/68	Male 2.63 ± 1.1865 Female 2.14 ± 0.76	/		/	/	5	/	
Wen et al, 2024^29^	48/52	12	0.55	0.75	5	1	1	75.8 ±15.5	158.6 ± 33.1
Rohit et al, 2023^26^	8/10	/	/		10	0.3	1	/	
Qin et al, 2023^24^	UDFU 31/34	12	/		/		0	233.88±23.2	432.42±79.97
	BDFU 26/34	12					3		
Liu et al, 2023^17^	LP 18/19	1.5±0.7	0.55±0.03	0.66±0.06	5	0	1	/	
	MP 37/39	1.4±0.7	0.56±0.03	0.65±0.05	6	0	2	/	
Jianda et al, 2023^13^	21/21	4.2±0.6	/		/		0	/	
Ou et al, 2022^21^	18/19	1	0.54±0.13	0.88±0.07	4.97±0.88	0.37±0.49	0	71.19±10.29	155.01±33.01
Fan et al, 2022^8^	20/21	1.6±0.8	0.45±0.13	0.56±0.12	5.25±0.35	3.53±0.37	1	/	
Chang et al, 2023^5^	13/13	0.85±0.26	0.7±0.2	0.9±0.1	5.2±1.2	1.4±1.0	0	/	
Yuan et al, 2021^32^	201/201	4	0.2	0.8	/		0	/	
Nie et al, 2021^20^	33/42	4.5	/		/		0	/	
	25/43	6.1					4		
Li et al, 2021^15^	13/13	<3	/		/		0	/	
Chen et al, 2020^6^	131/136	126/136 in 6 mo	/		/		4	/	
	98/137	56/137 in 6 mo					31		

Abbreviations: ABI, ankle brachial index; BDFU, bilateral diabetic foot ulcer; LP, learning phase; MP, mastery phase; UDFU, unilateral diabetic foot ulcer; VAS, visual analog scale; VEGF, vascular endothelial growth factor.

**Table 3.** Cross-Tabulation of Studies With Surgical Complications.

Outcomes	Surgical Complications			
	Pin-Site Infection	Tibial Fracture	Other/Additional Comments	Total Percentage of Any Type of Complications
Yu et al, 2024^31^	3	0	5 amputations	26
Wen et al, 2024^29^	–	–	1 amputation 2 ulcer recurrences	5.7
Rohit et al, 2023^26^	3	0	Infections improved with antibiotics and dressing 1 slowed healing time and a delay in consolidation managed by keeping frame on for 6 months 1 amputation	50
Qin et al, 2023^24^	–	–	3 amputations 2 ulcer recurrences	7.4
Liu et al, 2023^17^	–	–	3 drill bit fractures 2 of the fractures led to bone necrosis 3 cases of toe amputation 1 gastrointestinal bleed 1 myocardial infarction	11.7
Jianda et al, 2023^13^	1	–	Healed after debridement and dressing changes Two of 21 patients had superficial rupture at previous wounds, healed with dressing changes and antibiotics	14.3
Ou et al, 2022^21^	–	–	1 superficial surgical wound complication which healed with dressings	5.3
Fan et al, 2022^8^	–	–	1 amputation due to serious infection	4.8
Chang et al, 2023^5^	1	–	Healed after removal of pin and dressing changes	7.7
Yuan et al, 2021^32^	–	–	–	–
Nie et al, 2021^20^	–	–	–	–
Li et al, 2021^15^	1	–	1 mild displacement of osteotomized cortex due to early removal of frame Pin site treated with wound care and antibiotics	15.4
Chen et al, 2020^6^	3	2	Tibial fractures occurred 1 wk after external fixator removal and treated with closed reduction and external fixation and healed in 4 wk. Pin site treated with dressings 4 amputations 4 recurrences	9.6

## Discussion

### Healing Rate

Wound healing rate and time is an effective measurement at identifying the quality of treatment, especially when considering a newer surgery such as TTT. Complications such as infection and reduced blood supply can contribute to issues with healing of wounds, and a study demonstrated that healing rates in ulcers treated with only conservative treatment was as little as 40%.^10,18^ The case-control study by Nie et al^20^ demonstrates the benefits of TTT on healing rates. Their mean healing rate was 4.5 months in the TTT group in comparison to 6.1 months in the control (*P* = .02) as seen in Table 2 results of healing time. This study also demonstrated that TTT has a beneficial effect on ischemic ulcer healing rate, showing the healing rate in the TTT group to be 33/42 (79%) in comparison to the 25/43 (58%) in the control group (*P* = .04) shown in the ulcers healed results in Table 2.

Chen et al^6^ had similar results, where 126 of 136 patient’s (93%) ulcers healed in 6 months using TTT compared with only 56 of 137 (41%) in the control group (*P* < .001) as shown in Table 2. Other studies without a control comparison group had similar results to Chen et al and Nie et al^20^ in regards to healing rates, with Yu et al^31^ showing 93% of ulcers healing in 2.6 months for men and 2.1 months for female demonstrated in Table 2 results for ulcer healing time. Of the 724 cases of TTT, 681 patients had healed ulcers, meaning 94% of patients’ ulcers healed after the TTT surgery was performed. In comparison, of the 180 control cases throughout the studies, only 123 patients’ ulcers healed, resulting in only 68% of patients’ ulcers healing using more traditional methods. This percentage difference of 26% is attributed to TTT. Overall, healing times are also important to note, with TTT cases having an average of approximately 4.6 months taken for ulcers to heal whereas in the 2 control cohorts, 1 showed an average of 6.1 months for ulcers to heal whereas the other showed only 41% of ulcers healed in 6 months as reflected in Table 2. The limitation of this, however, is that many ulcers were not solely treated with TTT. Other more conventional treatments such as debridement, VSD, antibiotic bone cement, and medications were used in conjunction. This means the isolated effect of TTT on healing rates and time is not fully measured. It appears that TTT has a positive effect on healing rates and an evident reduction in the time to heal, demonstrated by the data shown from these studies.

### Vascular Endothelial Growth Factor

VEGF is an angiogenic factor that is known to promote growth cells derived from blood vessels such as arteries and veins.^9^ It is one of the many markers that demonstrate the rate and amount of angiogenesis in the body and hence is a surrogate marker to assess angiogenesis in limbs. Three articles, Qin et al,^24^ Ou et al,^21^ and Wen et al,^29^ assessed VEGF, all represented in the Table 2 results of VEGF; however, the change from pre- to postoperation was significant. In the study by Qin et al,^24^ a change from 233.88 ± 23.25 pg/mL to 432.42 ±79.97 pg/mL with *P* <.05 demonstrates the increase of this growth factor found in the blood. Ou et al^21^ demonstrated comparable results with 71.19±10.29 pg/mL to 155.01±33.01 pg/mL with a *P* <.05, and Wen et al^29^ had an increase from 75.8 ± 15.5 pg/mL to 158.6 ± 33.1 pg/mL. This increase in VEGF indicates a change in the level of angiogenesis occurring in the affected limb and demonstrates a potential mechanism for how TTT works in healing ulcers so efficiently. Correlation of TTT and VEGFs is an area that requires future research. Nonetheless, this significant increase in VEGFs following TTT is promising.

### ABI and VAS score

The ankle brachial index is relevant to this study as it is a repeatable ratio comparing blood pressure in the upper and lower limbs. Normal ratios are considered 1 to 1.4. A decreasing ratio is an objective measure of peripheral arterial disease.^27^ Improvement in ABI is a measurable indication that macrovascular blood flow is increasing to the affected limb, which indicates that TTT is effective in increasing angiogenesis and therefore beneficial to the healing of ulcers. The information regarding ABI was not uniformly discussed in the articles; however, 6 of 13 studies used ABI as an outcome to analyze the effect of TTT, with all relevant data presented in Table 2 under ABI. Liu et al^17^ demonstrated in both stages of the study a mean increase of 0.1 on the ABI scale (*P* < .001). Ou et al^21^ is another study where this ABI increase is pronounced, where the ABI changed from 0.54±0.13 to 0.88±0.07 after 180 days (*P* < .05). This improvement is indicative of how TTT has increased macrovascular blood flow to the affected peripheral limbs, promoting healing of peripheral ischemic ulcers.

VAS is a validated scale used to measure patient pain, and is still a valuable marker for comparison of pre- and post-surgery. This is important as intuitively, the mechanism of distracting bone via an external fixator device on the tibia is painful.^19^ Of the 6 articles, all demonstrated a reduction in VAS score, shown in Table 2.^5,8,17,21,26,29^ This is very positive for patients undergoing TTT as not only will the pain from their ulcer improve but the discomfort from the corticotomy and fixator does not appear to be significant.

### Limb Salvage

Limb salvage is a crucial outcome to assess when treating ischemic limb ulcers. A study carried out in the United Kingdom in 2012 indicated that the incidence of amputation in patients with diabetes alone was 2.51 per 1000 patients.^11^ Transtibial amputation in diabetes has been significantly linked to early mortality.^1^ Studies discussed limb salvage rates, with the percentage of amputations being generally low, with 6 studies reporting no amputations after the surgery, reflected in Table 2.^5,13,15,20,21,32^ This is a positive outcome as it demonstrates that TTT does not increase the risk of amputation. In one of the case-control studies, Chen et al,^6^ the comparison of amputation between the TTT group and the control demonstrates improvement in amputation rates. The TTT group had 4 amputations out of 136 patients (3%) compared with the 31 of 137 (23%) in the control group (*P* < .001). Out of the 724 cases using TTT in this systematic review, 18 cases resulted in amputation, which means less than 3% of patients who underwent the TTT surgery had an amputation. In those that had control groups, the rate of amputation was 35 of 180, which means 19% of cases had amputation after traditional treatments and surgery for ischemic ulcers.

### Complications and Risks

The results and outcomes of TTT are promising, with exclusively beneficial results seen within this systematic review. However, there are evidence of complications and risks associated with the surgery. The 2 main complications identified were pin-site infection and tibial shaft fracture. Of the 724 patients who underwent TTT, there were 12 pin site infections reported, reflected in Table 3 (<2%), all of which were treated with either dressing changes or oral antibiotics that allowed healing to be uneventful and prevented any deep infection occurring. It is important to note that the usual expected pin site infection requiring antibiotics in Ilizarov is about 20%, and this is shown to occur mostly in the second half of treatment when the frame is on for 3-6 months.^7^ In the TTT case, the frame is on for a shorter period of time and so the pin sites are less prone to infection, hence the reduced percentage. The moving pins are unicortical, on the medial face of the tibia, and do not irritate the thin soft tissue envelope with locomotion. The authors acknowledge that the rate of subclinical pin site colonization is likely to be much higher. There were a total of 2 tibial fractures reported (<1%), which healed with fracture care, detailed in Table 3.

The reported incidence of iatrogenic tibial fracture is very low with this technique but it remains an important aspect of informed consent for patients, and understanding of the higher risks associated with tibial fractures in regard to amputations in this patient group.

### Limitations

TTT is an emerging technique that does not have the same depth of literature as conventional ischemic ulcer treatments. The reported outcomes and the research protocols are inconsistent, leading to varied and different outcomes being studied. A second limitation is that the studies generally have a short follow-up time period, and so the data of healing and prevention of ulcer recurrence is limited; therefore, longer follow-up time is needed to observe patient outcomes more accurately. Another limitation of this study is that the majority of the articles had smaller population sizes, as well as most having a lack of a control group. The majority of the articles are Chinese, except for the singular article published from India. The disadvantage of this current data being only representative of a population based in one country is that it is therefore limiting with regard to broad outcomes that may be expected of an international study. TTT outcomes on international cohorts have yet to be studied; therefore, it may be less translatable to the wider population.

## Conclusion

TTT is an innovative solution to tackling both macrovascular and microvascular aspects of ischemic ulcers. It has been shown to improve surrogate markers of blood flow by significantly increasing vascular endothelial growth rate and ABI. More importantly, it has measurable benefit in helping to improve healing rate and limb salvage. The technique does not increase the VAS scores and indeed reduces pain associated with the ulcer and maybe is less painful than conventional surgical treatments. Complications and risks of the surgeries are not common; however, these risks do need to be taken into consideration when undertaking this surgery and consenting patients. Future research from a larger international cohort is needed so that the benefits and complications of TTT in a wider population can be better understood. It is not clear whether this technique is safe in the presence of osteoporosis, obesity, or commonly encountered skin changes seen in diabetes patients.

## Supplemental Material

sj-pdf-1-fai-10.1177_10711007251341312 – Supplemental material for A Systematic Review on Tibial Cortex Transverse Transport in the Treatment of Ischemic Ulcers of the Lower Limb Supplemental material, sj-pdf-1-fai-10.1177_10711007251341312 for A Systematic Review on Tibial Cortex Transverse Transport in the Treatment of Ischemic Ulcers of the Lower Limb by Abby Luxon, Anxhela Syziu, William D Harrison, Amirul Islam and Lyndon Mason in Foot & Ankle International
